# Editorial Board news and a celebration of paramedic research

**DOI:** 10.29045/14784726.2026.9.11.2.1

**Published:** 2026-09-01

**Authors:** Julia Williams

**Affiliations:** Editor-in-Chief, *British Paramedic Journal*

Paramedic research is thriving and the evidence has rarely been more compelling than it was in Birmingham on 16–17 June 2026, where the Royal College of Paramedics Research Conference brought together a remarkable gathering of researchers, clinicians, educators, students and leaders from across the profession and beyond. The conference was, by any measure, a resounding success. What was on display was not merely the breadth and quality of current research related to our profession but something even more significant. We experienced the products of a community of research with its own momentum, its own leadership, and, perhaps most encouragingly, a whole raft of new talent coming through. The depth of the programme, the confidence of the presenters and the quality of the discourse all pointed to a profession that is excited to demonstrate that research does make a difference. For those of us who have watched paramedicine’s research culture develop over many years, it was a genuinely inspiring occasion.

The *British Paramedic Journal* is proud to sit at the heart of that activity, providing a home for the knowledge that this community generates and a platform through which it can reach practitioners, educators, students and policymakers. As the discipline grows, so too must the Journal, and it is in that spirit that we are delighted to announce some important changes to our editorial team.

We are pleased to welcome Associate Professor Ursula Rolfe, Bournemouth University, UK, as our new Associate Editor. Ursula’s appointment marks a significant step in the Journal’s development, and we look forward enormously to working with her in shaping *BPJ*’s future direction.

We are also delighted to welcome four new members to the Editorial Board:

- Dr Jack Barrett, South East Coast Ambulance Service NHS Foundation Trust, UK.
- Samantha Laws, City St George’s University of London, UK.
- Dr Peter Phillips, Bournemouth University, UK.
- Dr Louise Reynolds, Australian Catholic University, Australia.

Together, they bring expertise spanning clinical practice, education and research, and they extend the Journal’s international reach.

At the same time, we say a fond farewell to James Brogan, Robert Gordon University, UK, who steps down from the Editorial Board after five years of dedicated service (2021–2026). James has been a committed and energetic presence on the team, and the Journal has benefitted greatly from his knowledge, enthusiasm and passion for the profession. We wish him every success as Consultant Editor for the *Journal of Paramedic Practice*.

These are genuinely exciting times for paramedicine. The Birmingham conference reminded us of how far the profession has come, but also how far it still needs to go, and the appointments announced here speak to how seriously we take the responsibility of supporting that journey.

Welcome to all our new colleagues, and thank you, James.

